# Bibliography

**Published:** 1889-07

**Authors:** 


### BIBLIOGRAPHY.

MERCK'S INDEX OF FINE CHEMICALS AND DRUGS, comprising a summary of whatever chemical products that are to-day considered as being useful in either medicine or technology, with average values and synonymes affixed. First American Edition. Published by E. Merck, manufacturing chemist, Darmstadt, Germany; American branch, 73 William Street, New York. Price, $1.00.

This is a very valuable addition to our list of technical books, and is well worth the price as a reference hand-book on chemical and pharmaceutical terms alone, to say nothing of its other valuable points. The common name for all drugs is given in connection with the chemical name, so that no one need fail in finding the article desired.

HOME GYMNASTICS FOR THE WELL AND SICK. By E. AUGERSTEIN, M.D., staff physician and superintendent of the gymnasiums of the city of Berlin, and G. ECKLER, head teacher of the Royal Institution for training teachers for gymnastics. Translated from the eighth German edition by Berthold Schlesinger, Brookline, Mass. Houghton, Mifflin & Co.: The Riverside Press, Cambridge, Mass.

The book is just what it purports to be,—viz., a guide for persons in search of healthy, rational exercise. There has been need of just such a work, that would indicate to all scientific rules for physical culture which did not involve the purchase of expensive apparatus. Too much stress cannot be laid upon the necessity for methodical exercise upon the part of all persons confined to sedentary occupations and to those who are convalescing. The book is fully illustrated, engravings being made to take the place of long, tedious descriptions. A complete index of all the figures found in the text is placed upon a separate plate for ready reference.

Part First gives a description of all the exercises advised, while Part Second prescribes their application in the case of invalids. We can fully recommend the book to all.

DENTAL SCIENCE: QUESTIONS AND ANSWERS ON DENTAL MATERIA MEDICA, DENTAL PHYSIOLOGY, DENTAL PATHOLOGY, AND THERAPEUTICS. By LUMAN C. INGERSOLL, A.M., D.D.S. Second Edition. The Wilmington Dental Manufacturing Co.: Philadelphia, Pa., 1889.

The fact that this book has reached its second edition shows the need of some such work, and justifies the author in his conclusions regarding this particular method of imparting instruction. The work does not pretend to be a treatise, but is more of a compendium, embracing the fundamental principles which underlie scientific dentistry as the author sees them. We were somewhat surprised, however, to see the reproduction of the author's ideas of the dual character of the pericementum after the somewhat rough handling this theory has received upon the part of scientific men in the profession. The chapter on Dental Pathology and Therapeutics should rather be termed Dental Practice, as it does not comprehend pathology. The author's ideas regarding suppuration and pus-formation are obsolete, so also are his theories regarding decay of the teeth. In the statement (page 106) that the direct connection of micro-organisms with decay has not been proven, the author denies the results of the work of Dr. Miller, or else is not acquainted with the painstaking researches of the latter gentleman: the germ theory of decay has been conclusively proven.

The book is in reality the condensed notes of Dr. Ingersoll, which were used by him while lecturing upon the subjects therein contained in the Dental Department of the University of Iowa, and reflect the observation and studies of this well-known teacher during many years of active practice.

Hygiene of the Nursery. By Louis Starr, M.D. Second Edition. P. Blakiston, Son & Co.: Philadelphia, 1889.

Dentists are often asked to recommend some work on the care of children's teeth. While the above book does not profess to be a manual on the subject, yet it contains some very good advice to mothers. The direction on page 147, as to how to remove the "dark-colored scum which forms at the junction of the tooth and gum," is thoroughly practical, but in this day of specialties seems a little out of place, because of the fact that with it is not coupled the further advice to consult a dentist in case of any tendency of the teeth to decay. The book is a good one and has gone through a second edition, and will form a valuable addition to your library.

Operative Dentistry. By Thomas Fillebrown, M.D., D.M.D. P. Blakiston, Son & Co.: Philadelphia, Pa., 1889.

This work, by the well-known author, forms one of a series of text-books which are in preparation under the auspices of the National Association of Dental Faculties. The series comprises a number of text-books, written by men whom the committee has thought best able to prepare them. The task assigned Dr. Fillebrown was not an easy one, since no special work on operative dentistry had ever been written, and because of the further fact that no strict line of division has ever been drawn between operative and mechanical dentistry. Some criticism has been passed upon the author because so much space has been devoted to crown- and bridge-work, some holding that the latter, if not the former also, should be classed with mechanical dentistry. Be that as it may, nearly every dentist who in years past has confined himself to what he considered operative dentistry has found himself forced in the last few years to take up crown- and bridge-work to the exclusion of large contour fillings, and the fact seems to justify the author's action of including them in a treatise on operative dentistry.

The work is embellished by 330 wood-engravings, a large proportion of which bear the trade-mark of the S. S. White Dental Manufacturing Company. The author has been somewhat severely criticised for allowing the trade-mark of instruments to have remained on the illustrations, not because of the special house favored, but because of the fact that the book necessarily becomes more or less an advertising catalogue of that house. In this instance it might seem to an outside observer that there were no other instrument-makers in America but the S. S. White Dental Manufacturing Company, or that the exclusive privilege of illustrating the book had been let to that company; either of which conclusions would be erroneous. The book, on the whole, is well gotten up, and reflects credit on the author and publishers.

DENTAL MEDICINE. By FERDINAND J. S. GORGAS, A.M., M.D., D.D.S.

By the same publishers as the above. It is a deservedly popular book, and has now reached its third edition. The author has brought the work up to date in regard to the latest methods of practice, and the use of antiseptics is fully set forth. No dental student, or dentist in practice who desires to be more than an accomplished artisan, can afford to be without some such work in his library. We can heartily recommend Gorgas's "Dental Medicine" as a hand-book especially adapted to dental practice.

For HARRIS'S PRINCIPLES AND PRACTICE OF DENTISTRY, revised and edited by Dr. Gorgas, and published by Blakiston also, we cannot give such hearty approval. It does seem that the day has passed for such works, which, as is stated by some reviewer, "savor of the 'Family Physician' without that book's adaptation to the family needs." There is much that is good in the work and also much that has not been digested,—an incongruous mass that needs rigid overhauling before it can be brought into shape for assimilation.

Geo. S. Davis, Detroit, Mich., sends three copies of the Physicians' Leisure Library Series,—THE RADICAL CURE OF HERNIA, by H. A. MARCY, A.M., M.D., LL.D.; BRIGHT'S DISEASE, by ALFRED L. LOOMIS, M.D.; and a translation of PROFESSOR ZIEMSSEN'S MEMOIR ON PULMONARY TUBERCULOSIS, by DAVID J. DOHERTY, A.M., M.D.

The mention of the names of these authors is alone sufficient to indicate that the subjects will be well handled. This deservedly popular series of brochures is issued monthly, at the very modest price of $2.50 per annum, or twenty-five cents per copy.

We are also in receipt of the INDEX CATALOGUE of the library of the Surgeon-General's Office, United States Army, Volume IX. A ponderous volume of over one thousand pages.

